# Transcatheter Aortic Valve Replacement in Special Population Groups

**DOI:** 10.31083/RCM45993

**Published:** 2025-11-25

**Authors:** Kameel Kassab, Karnav Modi, Christian Torres, Talal Asif

**Affiliations:** ^1^Division of Cardiology, Onvida Health, Yuma, AZ 85364, USA; ^2^Department of Internal Medicine, University of Missouri, Kansas City, MO 64112, USA; ^3^Division of Cardiology, University of Missouri, Kansas City, MO 64112, USA

**Keywords:** TAVR, transcatheter aortic valve replacement, special populations

## Abstract

Transcatheter aortic valve replacement (TAVR) has revolutionized the treatment landscape for severe symptomatic aortic stenosis among all surgical risk groups. Thus, following the expansion of TAVR use and constant improvements in TAVR platforms and implantation techniques, implementation has been extended to special population groups that were previously underrepresented in clinical trials. This review evaluates the role of TAVR in patients with unique clinical considerations, including those with active malignancies, psychiatric disorders, and advanced organ dysfunction. By examining current literature, we provide insights into the safety, efficacy, appropriateness, and specific challenges associated with TAVR in these patient groups.

## 1. Introduction

Severe aortic stenosis (AS) is a common valvular lesion with a prevalence of 12% in the elderly above the age of 75, with severe aortic stenosis occurring in 2–4% of this patient population [1]. AS leads to significant morbidity and mortality, particularly in elderly and comorbid patients. AS is a disease of aging and frequently co-exists with other comorbid conditions that are prevalent in the elderly, including dementia, organ dysfunction, and malignancies. Transcatheter aortic valve replacement (TAVR) provides a minimally invasive alternative to surgical aortic valve replacement (SAVR) in appropriately selected patients, offering promising outcomes for patients who are otherwise ineligible for traditional surgery. As TAVR indications and operator experience expand, it becomes essential to evaluate its implications in special populations that have traditionally been either excluded or under-represented in clinical trials. These groups are frequently encountered in clinical practice, complicating decision making and requiring a thorough heart team approach discussion that may involve multiple specialties outside the traditional cardiovascular and surgical services. This is reflected in the exclusion criteria from key randomized trials of the most commonly used TAVR platforms, including self-expandable and balloon-expandable valves. These trials frequently excluded groups such as those with cognitive dysfunction and advanced renal insufficiency and under-represented other conditions such as advanced lung or liver disease [2, 3, 4, 5, 6, 7]. There have been several reviews and registry data addressing TAVR experience and outcomes, mostly addressing underrepresented or excluded anatomic factors such as bicuspid aortic valves, rheumatic aortic stenosis, mixed aortic lesions, etc. [8].

In this review, we address several special populations with severe AS based on commonly excluded or underrepresented patient populations from pivotal TAVR trials that are frequently encountered in clinical practice. We describe the challenges associated with patient selection, pre-procedural work-up, intraprocedural management, and post-operative considerations. We chose commonly encountered groups in clinical practice for which data has been limited to small cohorts or series and provide a concise summary of the available literature. These include oncologic patients, patients with neuro-psychiatric conditions including dementia, and patients with commonly encountered advanced organ dysfunction.

## 2. TAVR in Patients With Active Oncologic Conditions

Patients with active cancers or a history of malignancy face unique challenges when undergoing TAVR. This group includes patients with a history of malignancy currently in remission, patients with active malignancy, and patients who were found to have malignancy on pre-TAVR computed tomography scan. In a meta-analysis of 38,695 patients involving 15 studies, the prevalence of current or previous cancer in patients undergoing TAVR was 19.8% [9]. Several studies evaluated the incidence of new malignancies on pre-TAVR work-up, particularly with computed tomography. Demirel *et al*. [10] reported in a cohort of 575 patients undergoing work-up for TAVR that incidental findings occurred in 63% of patients, among which 4.5% were confirmed malignancies. In an earlier cohort of 136 patients, Patel *et al*. [11] evaluated the prevalence of incidental findings on pre-TAVR computed tomography and their impact on survival and found an incidence of around 3% of confirmed malignancies. Symptomatic severe aortic stenosis has an average annual mortality of 15% to 25% [12] which portends a worse prognosis compared to many types and stages of cancer. This poses the clinical question of whether treating aortic stenosis first in certain cancer patients translates into improved outcomes. Several studies indicate that TAVR can be performed safely in selected oncology patients, providing symptomatic relief and improved quality of life [9, 13]. There are several factors to consider when evaluating this group for potential TAVR.

### 2.1 Preoperative Considerations

**Fig. 1.**
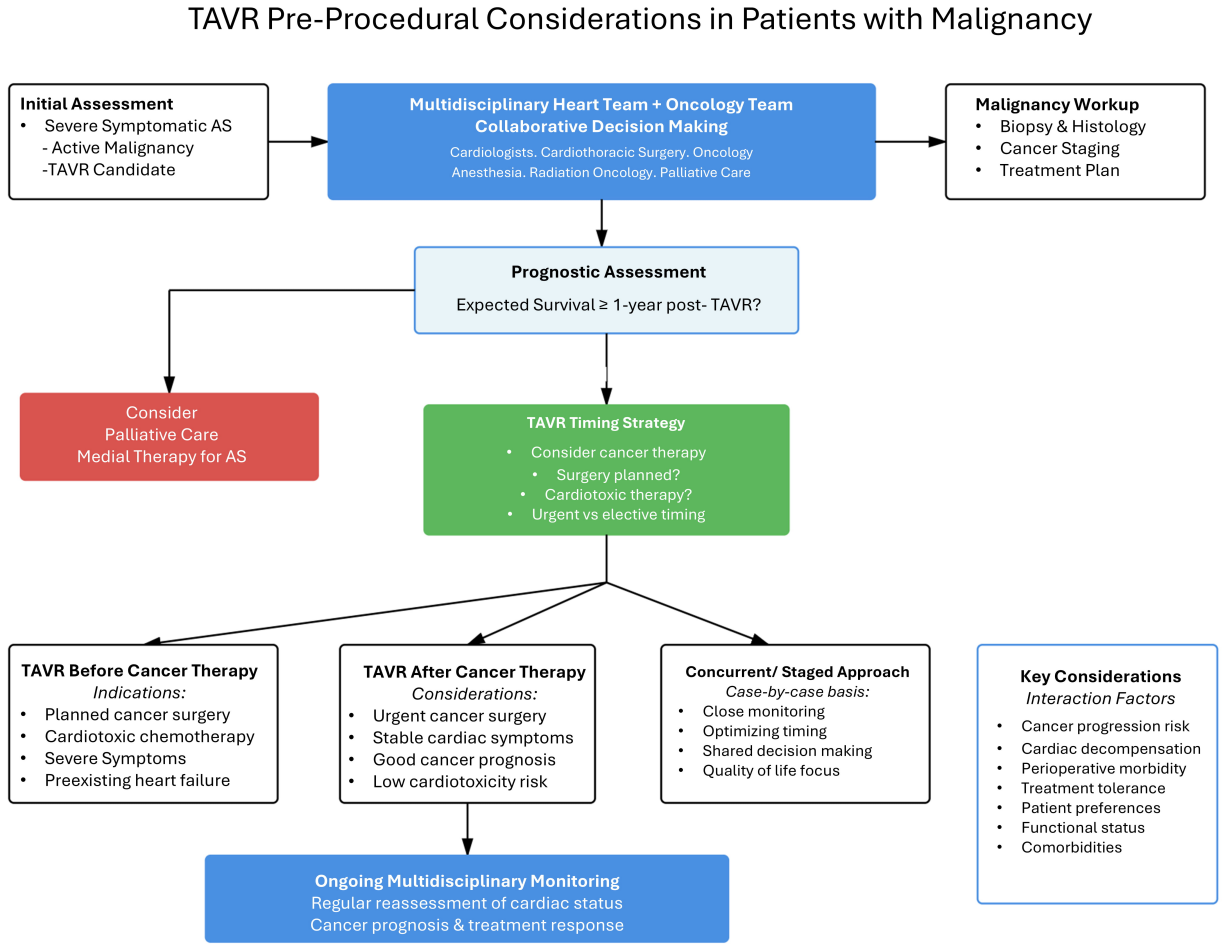
**Proposed pathways for workup of patients with active malignancy for TAVR**. TAVR, transcatheter aortic valve replacement; AS, aortic stenosis; ACC/AHA, American College of Cardiology and American Heart Association.

A comprehensive evaluation frequently starts with obtaining a biopsy for malignancy confirmation and typing, followed by cancer staging, prognostication, and finally formulating a treatment plan. Current practice recommendations from the American College of Cardiology and American Heart Association (ACC/AHA) advise against TAVR if patient’s expected survival is less than one year [12] after TAVR. This is a common exclusion criterion in most of the pivotal TAVR trials among all surgical risk groups. Yet, prognostication may be very challenging to determine, especially in newly diagnosed patients or in those who were found to have a malignancy on pre-TAVR workup. This usually requires close coordination between the heart team and the oncological services. Fig. 1 summarizes the proposed pathway for pre-procedural considerations for patients with active malignancy undergoing TAVR.

### 2.2 Interaction Between Aortic Stenosis and Cancer Therapies

There is limited data available on the optimal timing of TAVR in patients who are deemed to be procedural candidates. Timing also depends on whether patients need a surgical approach to cancer management, radiation, chemotherapy, or combination therapy. Frequently, patients with active malignancy undergoing chemotherapy or immunotherapies develop decompensations requiring hospitalizations for non-cardiac or cardiac events. Patients with severe symptomatic aortic stenosis may tolerate decompensation events and hospitalization poorly. It remains unclear whether performing TAVR in patients prior to initiation of treatments, particularly those with potential cardiac toxicity or patients with preexisting heart failure, would decrease morbidity of cancer treatments. Alternatively, delaying urgent therapies to perform TAVR may lead to cancer progression or worsening of patient prognosis. If patients require surgical resection, performing TAVR prior to surgical intervention may be reasonable to reduce operative morbidity. Fig. 1 provides an algorithm for workup of patients with active malignancy for TAVR.

### 2.3 Outcomes

Several observational studies have evaluated short- and long-term outcomes of cancer patients post-TAVR. Landes *et al*. [14] compared outcomes of 222 cancer patients who underwent TAVR to 2522 non-cancer patients. At the time of TAVR, 40% had stage 4 cancer, and periprocedural complications were comparable between the groups, including 30-day mortality. However, 1-year mortality was higher in cancer patients, with one-half of the deaths due to neoplasm. Progressive malignancy (stage III to IV) was a strong mortality predictor, whereas stage I and II cancer was not associated with higher mortality compared with no-cancer patients [14]. In an analysis of the Nationwide Readmissions Database for TAVR cases from 2012 to 2019, 122,573 patients undergoing TAVR were included in the analysis, of whom 8013 (6.5%) had active cancer. The authors found that the presence of active cancer was not associated with increased in-hospital mortality. There was, however, an increased risk of readmission at 30, 90, and 180 days after TAVR and increased risk of bleeding requiring transfusion at 30 days [13]. These findings have been previously observed in other observational studies where no significant difference in in-hospital mortality has been observed post-TAVR in patients with or without active malignancy [15]. There is additional variability in outcomes depending on the type of malignancy. Colon cancer has been observed to be related to more bleeding events post-TAVR, whereas breast cancer was associated with increased risk of pacemaker implantation post-TAVR [13]. The significance of the latter finding is unclear but may be related to the type of breast cancer chemotherapy and radiation therapies. Given these findings, it may be reasonable to perform close cardiac monitoring for breast cancer patients post-TAVR and minimize the use of antiplatelets or antithrombotics in patients with colon cancer post-TAVR. In a more recent meta-analysis of 9 studies including a total of 133,906 patients, of whom 9792 (7.3%) had active malignancy, Felix *et al*. [16] observed higher short- and long-term mortality rates in patients with active cancer undergoing TAVR, which were not driven by cardiovascular causes. Additionally, higher major bleeding was observed among cancer patients. There was no significant difference in cardiac, renal, and cerebral complications at follow-up ranging from 180 days to 10 years in patients undergoing TAVR with active cancer compared to no cancer [16]. Table 1 (Ref. [9, 13, 14, 15, 16, 17, 18]) summarizes key studies evaluating the outcomes of TAVR in patients with active cancer.

**Table 1.** **Summary of studies evaluating outcomes of cancer patients undergoing TAVR**.

Study (authors, year)	Type of study	Outcome/complication	Active cancer vs. no cancer (summary)	Key data/findings
Landes U *et al*., 2019 [14]	Retrospective cohort study	30 days and 1 year mortality.	Similar 30-day mortality. Higher 1 year mortality in active cancer.	Stage I and II cancer was not associated with mortality. Stage III to IV was associated with higher mortality.
Lind A *et al*., 2020 [17]	Retrospective cohort study	Periprocedural complications. 30-day mortality.	No difference in periprocedural complications or 30-day mortality.	HR 1.47 (95% CI: 1.16–1.87) for all-cause mortality at 10 years.
		10-year survival.	10-year survival significantly reduced in active cancer.	
Jain V *et al*., 2020 [15]	Retrospective cohort study (National Readmission Registry)	Post-procedural outcomes, in-hospital mortality and 30 day readmission.	Similar procedural and in-hospital mortality. Higher 30 day readmission.	No difference in all-cause in-hospital mortality (OR: 0.873 [95% CI: 0.715 to 1.066]; *p* = 0.183). Higher likelihood of 30-day readmission (OR: 1.21 [95% CI: 1.09 to 1.34]; *p * < 0.001).
Aikawa T *et al*., 2023 [13]	Retrospective cohort study (National Readmission Registry)	In‐hospital mortality. Bleeding requiring blood transfusion and readmissions at 30, 90, and 180 days after TAVR.	Similar short-term outcomes; Higher bleeding and readmission.	Active cancer was not associated with increased in‐hospital mortality (adjusted odds ratio [aOR], 1.06 [95% CI, 0.89–1.27]; *p* = 0.523).
				Active cancer was associated with an increased risk of readmission at 30, 90, and 180 days after TAVR and increased risk of bleeding requiring transfusion at 30 days.
Osawa T *et al*., 2024 [9]	Systematic review/meta-analysis	Short-term (in-hospital or 30-day) and long-term (≥12 months) mortality.	Lower risk of short-term mortality.	Patients with cancer had a lower risk of short-term mortality (odds ratio [OR] 0.69, 95 % confidence interval [CI] 0.61–0.77, *p * < 0.001) but a higher risk of long-term mortality (OR 1.54, 95% CI 1.35–1.76, *p * < 0.001).
			Higher risk of long-term mortality.	
				Patients with cancer had a lower incidence of postprocedural stroke and acute kidney injury but a higher incidence of pacemaker implantation than patients without cancer.
Felix N *et al*., 2024 [16]	Meta-analysis	Short term and long-term mortality, cardiovascular mortality and rates of bleeding.	Higher short and long-term non-cardiac mortality in active cancer.	Patients with active cancer had higher short- (OR 1.33; 95% CI 1.15–1.55; *p* < 0.001) and long-term mortality (OR 2.29; 95% CI 1.80–2.91; *p* < 0.001). Rates, not driven by cardiovascular mortality (OR 1.30; 95% CI 0.70–2.40; *p* = 0.40), and higher major bleeding rates (OR 1.66; 95% CI 1.15–2.42; *p* = 0.008).
Saberian P *et al*., 2025 [18]	Meta-analysis	In-hospital mortality, 30-day mortality, 1-year mortality, 2-year mortality, Procedural success.	No significant difference in short-term outcomes; Significantly higher long-term mortality in active cancer.	OR 1.17 (95% CI: 0.83–1.65) for in-hospital mortality; OR 0.93 (95% CI: 0.72–1.19) for 30-day mortality; OR 1.93 (95% CI: 1.45–2.56) for 1-year mortality; OR 2.65 (95% CI: 1.79–3.93) for 2-year mortality; Comparable procedural success between groups.

TAVR, transcatheter aortic valve replacement; HR, hazard ratio; CI, confidence interval.

These studies highlight that TAVR should be offered selectively to patients with severe symptomatic AS and active oncological conditions after close coordination with oncology teams. In appropriately selected patients, especially those with early-stage malignancies, TAVR is safe and carries a favorable prognosis.

## 3. TAVR in Patients With Psychiatric and Neuro-Psychiatric Disorders

Psychiatric conditions are highly prevalent in the United States, affecting approximately 18% of the US adult population annually [19]. These patients experience higher all-cause mortality rates (around 2.2-fold increase) and are less likely to receive guideline-directed medical therapy and have lower rates of undergoing invasive testing [20]. In a recent analysis of over 1.5 million severe AS patients, patients with depression, anxiety, bipolar disorder, substance use, and psychotic disorders had lower odds ratios for undergoing TAVR compared to patients with no psychiatric conditions. There was a significant link between psychiatric comorbidities and a lower likelihood of utilizing TAVR [19]. Patients with dementia and associated mood disorders represent another very challenging and prevalent patient population with severe AS and have been frequently excluded from pivotal TAVR trials.

### 3.1 Preoperative Considerations

There are major aspects that come into play when treating severe symptomatic aortic stenosis in this population.

(1) Symptomatology: Depending on the spectrum of severity, symptom assessment may be challenging, especially in patients with limited verbal skills who cannot clearly verbalize their symptoms. In such cases, severe AS may be diagnosed based on physical exam findings or incidentally found on diagnostic testing. Functional status assessment may also be challenging, especially in more sedentary patients.

(2) Cognitive function: Obtaining informed consent represents another difficulty in this population. The premise of informed consent is the ability of the patient to fully understand one’s condition, its implications on health, and verbalize understanding of any invasive testing or therapies that need to be implemented. Patients with dementia or severe psychiatric disorders who may have long life expectancy are often not able to give informed consent, and frequently power of attorney needs to become involved. Balancing longevity with quality of life presents complex decision-making challenges that place significant responsibility on surrogate decision-makers.

(3) Adherence to Treatment: Patients with psychiatric disorders may struggle with adherence to pre- and post-procedural care. This includes completing necessary pre-TAVR workup, attending multispecialty outpatient appointments, and following through with treatments for incidental findings that require attention prior to the index procedure. Post-TAVR follow-up with post-procedural testing, therapies, and outpatient appointments can also place time strain on these patients, especially during flares of mental illness.

### 3.2 Outcomes and Prognosis

Data on TAVR outcomes in patients with psychiatric and neuropsychiatric disease is limited. In a retrospective study using national inpatient sample data, the prevalence of mental illness among patients undergoing TAVR was 14.2%, including bipolar disorder, schizophrenia, other psychotic disorders, anxiety, or depression. Patients with mental illness had a higher risk of procedural complications, including myocardial infarction, pneumonia, major bleeding, blood transfusion, and acute renal failure compared to patients without mental illness [21]. In another study of nearly 22,000 patients undergoing TAVR, 13.5% met the definition of having serious mental illness, including schizophrenia, mood disorder, and anxiety disorders causing significant functional limitations. Patients undergoing TAVR with serious mental illness had longer length of stay and were less likely to be discharged to home [22].

Approximately one-third of patients with symptomatic severe aortic valve stenosis have some degree of cognitive impairment [23]. In a study of 57,805 patients undergoing TAVR, 5.0% had a diagnosis of dementia, and the authors found that TAVR was associated with an increased risk of bleeding requiring transfusion, discharge to a rehabilitation facility, in-hospital delirium, increased length of stay, but comparable in-hospital mortality in patients with dementia compared with patients without dementia [24].

Delirium is a serious complication affecting elderly patients undergoing TAVR and can frequently be under recognized. A meta-analysis has reported the post-TAVR prevalence of delirium of up to 24.9% among elderly patients over 60 years of age [25]. Delirium usually lasts for a few days to weeks but can last months in up to 20% of patients. There are multiple risk factors for delirium, including patient and procedural-related factors. Patient factors include advanced age, comorbid conditions, and baseline cognitive decline. Procedural factors include duration of the procedure, anesthetic burden used, and procedural complications. Additionally, hospitalization factors including immobilization, intensive care unit admission, constipation, and associated infections also play a detrimental role in post-procedural delirium [26]. Patients with delirium following TAVR have twice the length of hospital stay and approximately 3 times the risk of increased hospital readmissions and mortality within 180 days of the procedure. They are twice as likely to be admitted to a rehabilitation facility compared with their non-delirious counterparts [25, 27]. In a meta-analysis of 9 studies, acute renal injury, baseline carotid artery disease, and transapical transcatheter aortic valve implantation (TAVI) had the highest effect size on post-procedural delirium after TAVR. The impact of baseline cognitive impairment on post-operative delirium has been documented with an estimated odds ratio of 2.2 [25].

The impact of TAVR on baseline cognitive function and mood disorders has also been described. In an analysis of nearly 1300 patients who underwent TAVR in an Australian registry, 28% of patients reported symptoms of at least moderate anxiety or depression. After TAVR, 74% of these patients reported resolution of their symptoms, with around 8% of patients developing new-onset anxiety or depression. Predictors of new-onset anxiety or depression were non-home discharge, post-procedural stroke, myocardial infarction, or heart failure hospitalization [28]. The complex interaction between TAVR and cognitive function has been evaluated in several small-scale studies. In a cohort of 229 patients above the age of 70 undergoing TAVI, 37% of patients demonstrated improvement in cognitive function as measured by mini-mental status examination, with patients demonstrating largest benefit showing the smallest valve area before TAVR. Notably, 12.7% of patients showed cognitive decline after TAVR [29]. This finding was also observed in an earlier cohort by Ghanem *et al*. [30], who reported that 9% of patients experience cognitive decline after TAVR. There are no clear predictors of cognitive decline after TAVR that were identified in both studies other than advanced age and possibly post-procedural delirium, which was observed in a quarter of patients undergoing TAVR in the former study [29]. Although post-procedural strokes, which occur in about 2.3% of TAVR patients [31], could provide an explanation, they were not found to be a predictive factor in either study. Another potential mechanism could potentially involve periprocedural microembolization phenomena, which have been thoroughly described post-TAVR yet have not yet been clearly associated with neurologic events [32, 33]. On the other hand, one of the basic proposed hypotheses for post-TAVR improvement in cognitive function is linked to improving cerebral blood flow. In a study of 148 patients who underwent TAVR, authors demonstrated significant improvement of cardiac output and cerebral blood flow at 3 months post-procedure. Global cognitive functioning also significantly increased, with the most prominent increase in patients exhibiting worst baseline cognitive functioning. Interestingly, improvement in cerebral blood flow had no impact on improvement of global cognitive function, and around 22% of patients had cognitive decline [23]. Larger-scale studies would be needed to better elucidate the relationship between TAVR and change in cognitive function and define the major predictors of both improvement and decline in cognition post-TAVR.

**Fig. 2.**
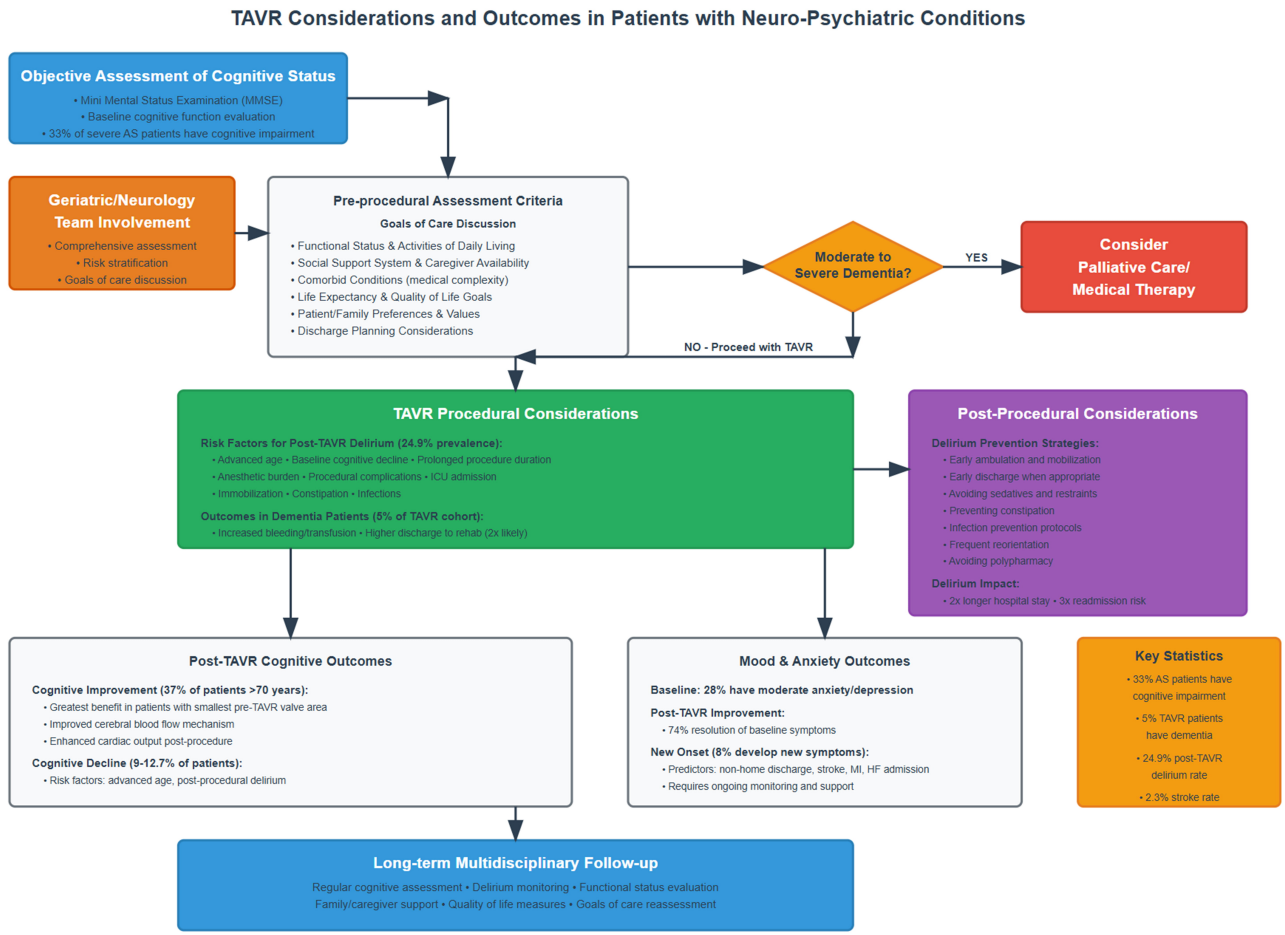
**Proposed pathway and summary of TAVR considerations and outcomes in patients with neuropsychiatric conditions undergoing TAVR**. TAVR, transcatheter aortic valve replacement; AS, aortic stenosis.

Available literature highlights the challenges psychiatric and neuropsychiatric conditions can have on patients with severe AS, including both pre-operatively and during recovery periods. Yet, procedural outcomes in general do not appear to differ significantly compared to the general population undergoing TAVR. Fig. 2 summarizes pre- and post-procedural considerations for patients with neuropsychiatric disorders undergoing TAVR.

## 4. TAVR in Patients With Advanced Organ Dysfunction

**Fig. 3.**
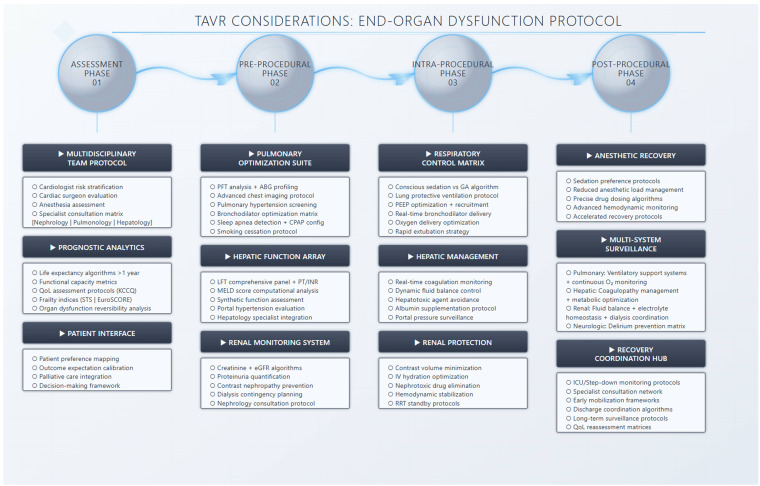
**Preprocedural, intraprocedural and post procedural assessment of patients with chronic lung disease, chronic liver disease, and chronic kidney disease undergoing TAVR**. TAVR, transcatheter aortic valve replacement; KCCQ, Kansas city cardiomyopathy questionnaire; STS, Society of Thoracic Surgeons; PFT, pulmonary function testing; ABG, arterial blood gas; MELD, Model for End-Stage Liver Disease; eGFR, estimated glomerular filtration rate; GA, general anesthesia; ICU, intensive care unit; LFT, liver function test; PT/INR, prothrombin time/international normalized ratio; PEEP, positive end-expiratory pressure; RRT, registered respiratory therapist.

Advanced organ dysfunction represents another subgroup of patients that is routinely encountered in clinical practice yet has been generally underrepresented in clinical trials. Advanced chronic kidney disease, dialysis patients, severe pulmonary hypertension patients, and Child C cirrhosis patients have been excluded from the pivotal CoreValve and PARTNER trials [2, 3, 4, 5, 6, 7]. Although there are other groups that have also been excluded, including GI bleeding, thrombocytopenia, leukopenia, etc., in this review we chose the most commonly encountered advanced organ dysfunction groups and discuss pre-procedural, procedural, and post-operative considerations based on the available literature. Fig. 3 summarizes pre-, intra-, and post-procedural considerations of patients within these groups undergoing TAVR.

### 4.1 TAVR in Patients With Advanced Lung Disease

Advanced lung disease incorporates various lung conditions, including chronic obstructive pulmonary disease (COPD) and interstitial lung disease (ILD). Frequently, these groups have associated pulmonary hypertension, the severity and cardiovascular consequences of which could vary depending on the severity of the pulmonary condition. These patients often present with compounded risks due to the coexistence of compromised pulmonary function alongside cardiovascular pathology with frequently overlapping symptomatology. Lung disease prevalence increases with age and is frequently encountered in aortic stenosis patients. It is estimated that 16% to 43% of patients with AS undergoing TAVR have comorbid COPD [34]. TAVR has even been described in patients undergoing workup for lung transplant and in lung transplant patients with favorable outcomes [35]. As such, their management requires meticulous preoperative evaluation, carefully devised perioperative strategies, and vigilant postoperative care to ensure optimal outcomes.

### 4.2 Perioperative Considerations

The perioperative assessment of patients with advanced lung disease undergoing TAVR frequently necessitates input from pulmonologists or anesthesiologists during heart team discussions. Comprehensive pulmonary function testing (PFT), including spirometry, diffusion capacity assessment, and arterial blood gas (ABG) analysis, helps determine the severity and functional implications of lung disease. Frequently, lung disease is discovered on pre-TAVR computed tomography (CT) scans, with the most frequent incidental finding being pulmonary nodules [11, 36]. Alternatively, other conditions including emphysema or pulmonary fibrosis are occasionally encountered and frequently require additional imaging modalities such as high-resolution computed tomography (HRCT) scans, which can offer valuable insights into the anatomical and pathological intricacies influencing patient selection and procedural planning. In a cohort of 373 patients with a median age of 84 years, fibrosis and emphysema were present in 66 (17.7%) and 95 (25.5%) patients, respectively. Fibrosis as a dichotomous variable was independently associated with the composite of death and readmission hazard ratio [HR], 1.54; *p* = 0.030, and CT evidence of fibrosis was a powerful predictor of adverse events [37]. Optimizing pulmonary status preoperatively is critical for mitigating perioperative risks. Strategies may include smoking cessation interventions, initiation of bronchodilators, inhaled corticosteroids, and targeted pulmonary rehabilitation programs. These interventions aim to enhance respiratory mechanics, reduce airway hyperreactivity, and optimize oxygenation before TAVR, thereby potentially reducing the incidence of perioperative respiratory complications. Procedural techniques and anesthetic management during TAVR also significantly influence outcomes. Traditionally, general anesthesia (GA) was frequently the preferred choice of anesthesia in early TAVR experience [38]. However, over the last 10 years, as the TAVR experience grew with more shift towards lower-risk patients, femoral and smaller bore access, safer valve platforms, shorter duration of procedures, and less reliance on transesophageal echocardiography, the mode of anesthesia has shifted towards monitored anesthesia care (MAC) [39]. In a meta-analysis of 7 observational studies including 1542 patients compared to GA, MAC was associated with a shorter hospital stay (–3.0 days (–5.0 to –1.0); *p* = 0.004) and a shorter procedure time (MD –36.3 minutes (–58.0 to –15.0 minutes); *p* < 0.001). Overall 30-day mortality, cardiac and procedure-related mortality was not different [40]. These findings were replicated in a more recent retrospective cohort study where 154 GA patients and 154 MAC patients underwent propensity matching. There were no differences in the safety outcomes of in-hospital or 30-day mortality, in-hospital or 30-day stroke, cardiac arrest, and need for permanent pacemaker between GA and MAC groups. There was a significant reduction in composite bleeding/vascular events in the MAC group (8.4% vs 19.5%, *p* < 0.01), reduced length of hospitalization and intensive care unit (ICU) stay, and higher likelihood of discharge to home [41]. In patients with severe lung disease undergoing TAVR, however, mode of anesthesia cannot always be generalized and is highly dependent on the presence of significant restriction or obstruction and is chosen on an individualized basis. In a more recent small cohort of 72 patients with severe COPD undergoing TAVR, the use of GA compared with MAC was associated with higher incidences of respiratory-related complications and longer intensive care unit length of stay [42]. Finally, careful attention to fluid management is essential to prevent volume overload and pulmonary edema, a critical consideration given the limited respiratory reserve in this patient population.

### 4.3 Outcomes and Prognosis

Multiple studies have demonstrated various outcomes in patients with chronic lung disease (CLD) undergoing TAVR. In an analysis from the PARTNER trial, patients with CLD who underwent TAVR demonstrated higher 1-year all-cause mortality compared to those without CLD (23.4% vs. 19.6%, *p* = 0.02). However, among patients with CLD, those treated with TAVR had significantly lower 2-year all-cause mortality than those who received standard therapy (52.0% vs. 69.6%, *p* = 0.04). Furthermore, within the CLD cohort, poor mobility—defined by a 6-minute walk distance of <50 m—and oxygen dependency were identified as independent predictors of worse outcomes [43]. In a study by Mok *et al*. [44], patients with COPD who underwent TAVR had significantly lower 1-year survival compared to those without COPD (70.6% vs. 84.5%, *p* = 0.008), and COPD was identified as an independent predictor of mortality. Despite this, COPD patients still demonstrated improvements in functional status following TAVR, as measured by NYHA functional class, Duke Activity Status Index, and the 6-minute walk test (6MWT). The study also found that reduced 6MWT distance and poorer baseline spirometry were associated with higher post-TAVR mortality. Crestanello *et al*. [45] reported similar findings from the CoreValve US Pivotal trial. The authors reported no difference in mortality at 30 days, but higher mortality in those with moderate to severe CLD at both 1 year (28.1% moderate, 26.9% severe vs. 19.2% non-CLD; *p* = 0.030) and 3 years (53.0% moderate, 51.9% severe vs. 37.7% non-CLD; *p* < 0.001) following TAVR. While over 80% of CLD patients experienced improvements in NYHA functional class and Kansas City Cardiomyopathy Questionnaire overall summary score (KCCQ-OS) scores at both 1 and 3 years, only a subset achieved a favorable overall health benefit—43.3% at 1 year and just 22.5% at 3 years. Similar findings were reported by Doldi *et al*. [34], who evaluated a retrospective cohort of 3408 patients who underwent TAVR, with 553 patients (16.2%) having comorbid COPD. Estimated 2-year survival rates were 69.2% (95% CI: 65%–74%) for COPD vs. 79.7% (95% CI: 76%–84%) for no-COPD patients. The presence of COPD was associated with a significantly increased mortality after TAVR (HR: 1.74; 95% CI: 1.36–2.22; *p* < 0.001). Authors also demonstrated that GOLD severity stage was associated with increased mortality (*p* < 0.001 by log-rank test). Hence, COPD is an independent predictor for mortality in TAVR patients. The authors observed that TAVR reduced symptoms in COPD patients, with more reduction in GOLD1/2 compared to GOLD 3/4 [34]. Table 2 (Ref. [34, 43, 44, 45, 46, 47]) summarizes studies evaluating TAVR outcomes in patients with CLD.

**Table 2.** **Summary of studies evaluating outcomes of Patients with CLD undergoing TAVR**.

Study (authors, year)	Study design/population	Main outcomes	Lung disease vs. no lung disease	Key findings
Mok M *et al*., 2013 [44]	Single-center retrospective cohort	1-year survival; functional status; predictors of mortality	1-year survival: 70.6% (COPD) vs. 84.5% (no COPD), *p* = 0.008	COPD was an independent predictor of mortality. COPD patients had significant improvement in NYHA class, DASI, and 6MWT post-TAVR. Lower 6MWT and poor spirometry predicted higher mortality.
Dvir D *et al*., 2014 [43]	Analysis from PARTNER trial; patients with severe AS and CLD	1-year and 2-year all-cause mortality; predictors of outcome	1-year mortality: 23.4% (CLD) vs. 19.6% (no CLD), *p* = 0.02, 2-year mortality (CLD, TAVR vs. standard therapy): 52.0% vs 69.6%, *p* = 0.04	CLD is associated with higher 1-year mortality after TAVR. TAVR improved 2-year survival vs. standard therapy in CLD. Poor mobility and oxygen dependency predicted worse outcomes.
Suri RM *et al*., 2015 [46]	STS/ACC TVT Registry analysis	1-year mortality	1-year mortality: 32.3% (severe CLD) vs. 21% (no severe CLD), vs. 25.5% (moderate CLD)	Moderate or severe CLD is associated with an increased risk of death to 1-year after TAVR. In patients with severe CLD, the risk of death appears to be similar with either transapical or transaortic alternate-access approaches.
Liao YB *et al*., 2016 [47]	Systematic review and meta-analysis; 28 studies	Short- and long-term mortality; complications	COPD negatively impacted both short-term and long-term all-cause survival (30 days: odds ratio [OR] 1.43, 95% CI, 1.14–1.79; >2 years: hazard ratio [HR], 1.34, 95% CI, 1.12–1.61)	COPD is common among patients undergoing TAVI, and COPD impacts both short- and long-term survival.
Crestanello JA *et al*., 2017 [45]	CoreValve US Pivotal Trial Analysis	1-year, and 3-year mortality; functional status assessment	All-cause mortality was higher in patients with moderate and severe CLD at 1 year (19.6% mild, 28.1% moderate, 26.9% severe CLD vs. 19.2% non-CLD; *p* = 0.030) and 3 years (44.8% mild, 53.0% moderate, 51.9% severe vs. 37.7% non-CLD; *p * < 0.001)	CLD is associated with higher 1- and 3-year mortality. >80% of CLD patients improved in NYHA and KCCQ-OS.
Doldi P *et al*., 2022 [34]	Retrospective single center cohort	2-year survival; mortality predictors	2-year survival: 69.2% (COPD) vs. 79.7% (no COPD), *p * < 0.001 HR for mortality: 1.74 (95% CI: 1.36–2.22), *p * < 0.001	COPD independently associated with increased mortality after TAVR. Higher GOLD stage = higher mortality. TAVR reduced symptoms in COPD, especially GOLD 1/2.

COPD, chronic obstructive lung disease; NYHA, New York heart association; DASI, duke activity status index; 6MWT, 6 minute walk test; AS, aortic stenosis; CLD, chronic lung disease; STS/ACC TVT, Society of Thoracic Surgeons/American College of Cardiology Transcatheter Valve Therapy; TAVI, transcatheter aortic valve implantation; CI, confidence interval; KCCQ-OS, Kansas city cardiomyopathy questionnaire overall summary score; GOLD, global initiative for chronic obstructive lung disease.

Several studies have evaluated the predictive value of pulmonary function testing and the presence of CLD on outcomes post-TAVR. Objective assessments of PFT can provide valuable insight into prognosis and guide clinical decision-making in this population. In a study by Pino *et al*. [48], increased alveolar-arterial (A-a) gradient, elevated PCO_2_, and reduced PO_2_ were associated with a higher risk of 30-day mortality. Interestingly, neither a diagnosis of COPD or CLD nor PFT parameters were significantly associated with 30-day or 1-year mortality. Conversely, Henn *et al*. [49] found that even patients without a known preoperative diagnosis of COPD or CLD often had abnormal PFTs, and moderate to severe lung disease—predominantly identified through PFTs—was an independent predictor of mortality following TAVR. Similarly, Lin *et al*. [37] demonstrated that CT evidence of pulmonary fibrosis was independently associated with increased mortality and readmission rates, particularly in patients without a prior diagnosis of CLD, while emphysema did not emerge as a significant predictor of outcomes.

Collectively, these studies highlight that patients with advanced lung disease undergoing TAVR represent a high-risk group with generally poorer postoperative outcomes. Nonetheless, when appropriately selected, many of these patients experience meaningful improvement in functional capacity and survival compared to those receiving standard medical therapy. These findings emphasize the importance of thorough preoperative evaluation, incorporating a multidisciplinary team approach and aligning decisions with patient preferences, to optimize candidate selection for TAVR in the setting of advanced pulmonary disease. Objective assessments—such as PFTs, HRCT, ABG analysis, and 6MWT distance—are instrumental in guiding this decision-making process.

## 5. TAVR in Patients With Advanced Liver Disease

Patients with chronic liver disease represent a high-risk cohort for surgical intervention. The 30-day mortality risk following cardiac surgery is 9% in patients with liver cirrhosis Child-Pugh class A, 37.7% in patients with class B, and 52% in patients with class C [50]. In treatment of patients with severe symptomatic AS, TAVR has emerged as a lower-risk alternative for patients with liver cirrhosis as a bridge to facilitate liver transplant [51, 52] and as a therapy to help mitigate morbidity and mortality. Patients with chronic liver dysfunction, particularly those with cirrhosis, present with a unique risk profile that influences procedural planning, perioperative management, and long-term outcomes following TAVR. Although some patients with liver disease have been included in clinical trials of TAVR platforms (2–4% liver disease prevalence in PARTNER 1 trial [53]), most studies excluded patients with Child C cirrhosis.

### 5.1 Perioperative Considerations

A critical step in evaluating patients with coexisting aortic stenosis and liver disease is the accurate assessment of hepatic function. The Child-Pugh classification and Model for End-Stage Liver Disease (MELD) scores are widely used tools to stratify liver disease severity and predict perioperative risk. Prognostic utility of combination scores has not been validated in patients with liver disease undergoing TAVR. Patients with Child-Pugh Class C or MELD scores greater than 15 are generally considered to have significantly increased operative risk due to poor hepatic reserve, coagulopathy, and hemodynamic instability [54, 55]. Additionally, these patients often have comorbidities such as hepatorenal syndrome, hepatic encephalopathy, and malnutrition that further compound their procedural risk. As a result, multidisciplinary heart team involvement incorporating hepatology services is essential in weighing the risks and benefits of TAVR in this population.

### 5.2 Procedural Considerations

Patients with cirrhosis are at increased risk of complications from both anesthesia and procedural perspectives. In general, conscious sedation (CS) is increasingly favored over GA due to its association with improved outcomes, including lower all-cause mortality, reduced procedural complications, shorter hospital stays, and decreased need for postoperative ventilation [38]. This can be especially beneficial in this cohort of liver disease patients to reduce hemodynamic instability and hence further hepatic insult from GA. However, certain anatomical or procedural constraints—such as the need for alternative access with surgical cutdown due to hostile iliofemoral artery disease—can necessitate general anesthesia [56] and thereby increase perioperative risk in this cohort. Moreover, patients with advanced liver disease often have fragile vascular structures and higher bleeding tendency secondary to coagulopathy and platelet dysfunction. This can have a significant impact on access site choice and closure, intraprocedural anticoagulation management, and post-procedural antiplatelet management. Access site choice is one of the most important considerations in this patient group due to significant coagulopathy. Percutaneous transfemoral access remains the favorable access site with the least complication risk. With the advent of lithotripsy as a tool to navigate iliofemoral disease, it is feasible to perform in up to 95% of cases in contemporary practice [57]. Alternative access, however, is required in select patients with hostile iliofemoral disease or when the femoral sizing is prohibitive. Although studies specifically comparing alternative access sites in patients with chronic liver disease are lacking, for patients with coagulopathy, choosing percutaneous alternative access may mitigate bleeding risk and may facilitate conscious sedation compared to higher-risk GA. Percutaneous axillary access could be performed by experienced operators and may reduce bleeding risk and vascular complications typically associated with surgical subclavian cutdown [57, 58]. More recently, transcaval access has emerged as another percutaneous option with favorable outcomes, including lower risk of acute renal injury, shorter duration of hospital stays, and more favorable neurovascular outcomes compared to other alternative access sites [57].

### 5.3 Outcomes and Prognosis

Several studies have reported comparable outcomes for patients with advanced liver disease undergoing TAVR. In a multicenter study involving Europe and Canada by Tirado-Conte *et al*. [59], the authors reported similar cardiovascular mortality between those with and without liver disease at 2-year follow-up and higher non-cardiac mortality in the liver disease group, with Child-Pugh class B or C and renal impairment being independent predictors of mortality. In another study by Lee *et al*. [60] utilizing the National Inpatient Sample (NIS), the authors reported no difference in mortality for those with and without chronic liver disease (cirrhosis, hepatitis B/C, alcoholic/fatty/nonspecific liver disease). In a study by Yassin *et al*. [61] utilizing the NIS, the authors reported that patients with advanced liver disease undergoing TAVR had no significant increase in the risk of in-hospital mortality or post-procedural complications. Additionally, Annie *et al*. [62], in their analysis using the TriNetX database, reported that in those with cirrhosis, the TAVR group had a lower mortality rate compared with the no-TAVR group, suggesting a mortality benefit associated with TAVR in patients with liver cirrhosis. Collectively, this suggests that TAVR is a feasible treatment for patients with liver disease, exhibiting comparable in-hospital outcomes to those without liver disease. It has been shown to be safe and feasible in this subset of patients, particularly for patients deemed high-risk surgical candidates who have been turned down for SAVR. Hence, careful patient selection and multidisciplinary care can lead to favorable results in selected individuals. Table 3 (Ref. [59, 60, 61, 62, 63, 64, 65]) summarizes studies evaluating TAVR outcomes in patients with chronic liver disease.

**Table 3.** **Summary of studies evaluating outcomes of patients with liver disease undergoing TAVR**.

Study (authors, year)	Study design/population	Main outcomes assessed	Liver disease vs. no liver disease	Key findings
Yassin *et al*. (2018) [61]	Retrospective Analysis	In hospital mortality	Patients with cirrhosis who underwent TAVR:	Similar inpatient mortality and complication rates.
	National Inpatient Sample, 2011–2014; TAVR in patients with and without cirrhosis.	Complications	In-hospital mortality OR 1.12, 95% CI 0.59–2.10, *p* = 0.734. Vascular complications OR 0.47, 95% CI 0.23 to 0.98, *p* = 0.043	Cirrhotic patients were less likely to develop vascular complications and to require a pacemaker however more likely to have non-routine home discharges.
		LOS		
			Pacemaker during index admission: Cirrhosis 6.23% vs. 10.77%.	
			Nonroutine discharge: OR 1.50, 95% CI 1.15 to 1.96, *p* = 0.003.	
Lak *et al*. (2021) [63]	Retrospective, single center, 2015–2018.	1-year mortality, 30-day pacemaker	Cirrhosis vs no Cirrhosis	Patients with severe AS with concomitant liver cirrhosis who underwent TAVI demonstrated comparable outcomes to their noncirrhotic counterparts.
			1-year mortality (12% vs. 12%, *p* = 1)	
	TAVR with SAPIEN 3	HF readmission	30-day new pacemaker rate (6% vs. 9%, *p* = 0.85)	
	Cirrhosis (n = 32) vs. no cirrhosis (n = 996).	MACCE	30-day and 1-year readmission rate for heart failure (11% vs. 1% and 12% vs 5%, *p* = 0.12)	
			1-year major adverse cardiac and cerebrovascular event rate (15% vs. 14%, *p* = 0.98).	
Tirado-Conte *et al*. (2018) [59]	Multicenter, propensity-matched, 12 centers; TAVR with (n = 114) vs. without liver disease (n = 114).	In-hospital/2-year mortality, complications	In-hospital mortality (9.4% vs. 6.5%; *p* = 0.433).	In-hospital mortality and complications were similar between matched groups except for acute kidney injury which was more common in liver disease group. Patients with CKD and more advanced liver disease are at a higher risk.
			Noncardiac mortality 2 years (26.4% vs. 14.8%; *p* = 0.034)	
		Predictors of mortality	Lower glomerular filtration rate (hazard ratio, 1.10, for each decrease of 5 mL/min in estimated glomerular filtration rate; 95% confidence interval, 1.03–1.17; *p* = 0.005)	
			Child-Pugh class B or C (hazard ratio, 3.11; 95% confidence interval, 1.47–6.56; *p* = 0.003).	
Ma *et al*. (2020) [64]	Meta-analysis of 5 studies	Post-TAVR outcomes	In patients with chronic liver disease undergoing TAVR:	TAVI outcomes were comparable between the patients with or without chronic liver disease, lower rate of pacemaker implantation in the patients with chronic liver disease.
	TAVR with vs. without chronic liver disease.		In-hospital mortality (OR, 1.39 [0.68–2.85], *p* = 0.36)	
			Pacemaker implantation (OR, 0.49 [0.27–0.87], *p* = 0.02).	
	Total TAVI 1476 patients			
	600 with liver disease.			
Jiang *et al*. (2022) [65]	Meta-analysis, 21 studies	Short-term and 1–2-year mortality	Hepatic insufficiency short-term (OR 1.62, 95% CI 1.18–2.21).	Hepatic insufficiency was associated with higher short-term and 1–2 year mortality.
	TAVR with vs. without hepatic insufficiency.		1–2 year mortality (HR 1.64, 95% CI 1.42–1.89).	
Lee *et al*. (2021) [60]	Retrospective, National Inpatient Sample, 2011–2017; TAVR with (n = 606) vs. without chronic liver disease (n = 1818).	In-hospital mortality, LOS, complications	TAVR liver disease vs. No liver Disease.	Similar in hospital mortality, complications, LOS and costs.
			In-Hospital mortality (2.81% vs. 2.75% OR 1.02 95% CI 0.58–1.78)	
			Length of stay (6.29 vs. 6.44 days, *p* = 0.29).	
			Costs ($228,415 vs. $226,682, *p* = 0.048).	
Annie *et al*. (2023) [62]	Retrospective cohort study. TriNetX multi-center database with propensity score matching. Patients with severe aortic stenosis and liver cirrhosis: TAVR group = 1283 No-TAVR group = 19,210	All-cause mortality at 365 days (1-year mortality)	Study compares TAVR vs. no-TAVR, all within cirrhotics. 1-year all-cause mortality: significantly lower with TAVR (22.5%) vs. no TAVR (34.8%). No data on procedural or postoperative complications.	The TAVR group had significantly lower 1-year mortality: 22.5% vs. 34.8% (*p * < 0.0001), confirmed by log-rank test.

TAVR, transcatheter Aortic Valve Replacement; NIS, National Inpatient Sample; LOS, Length of Stay; AKI, acute kidney injury; OR, odds ratio; CI, confidence interval; HR, hazard ratio; AS, aortic stenosis; MACCE, major adverse cardiac and cerebrovascular events; CKD, chronic kidney disease; HF, heart failure.

## 6. TAVR in Patients With Advanced Kidney Disease 

Chronic kidney disease (CKD) is a prevalent comorbidity in patients undergoing TAVR and poses distinct challenges in both preoperative risk assessment and postoperative care. The prevalence and accelerated progression of AS in CKD patients can be attributed to a pro-calcific environment driven by disturbances in mineral metabolism, chronic inflammation, and the accumulation of uremic toxins [66]. Conversely, AS-related heart failure can further compromise renal function through the development of cardiorenal syndrome [67]. This bidirectional relationship creates a vicious cycle in which worsening renal dysfunction exacerbates cardiac impairment and accelerates the progression of AS [68]. TAVR presents an opportunity to interrupt this cycle by alleviating the hemodynamic burden of severe AS, potentially leading to improvements in renal function through both decongestion and improvement of forward flow. However, the procedure carries its own set of risks that may negatively impact kidney function. These include exposure to iodinated contrast, rapid ventricular pacing during valve deployment—which temporarily reduces cardiac output—and the risk of arterial microembolization from the TAVR delivery system or valve debris [69]. As TAVR continues to be offered to increasingly complex and high-risk patient populations, including those with advanced CKD and end-stage renal disease (ESRD), it becomes essential to carefully consider its impact on both procedural outcomes and long-term prognosis in this vulnerable group.

### 6.1 Preoperative Risk Assessment

Patients with advanced CKD, especially those on chronic dialysis, are at significantly increased risk of perioperative complications, including bleeding, infection, and hemodynamic instability [70]. Moreover, these patients often exhibit vascular calcification, impaired platelet function, anemia, and altered drug pharmacokinetics, which further complicate both the procedure and recovery. As such, a comprehensive, multidisciplinary preoperative assessment incorporating nephrologists with the standard heart team is essential to determine candidacy and optimize care. Pre-procedure imaging and vascular evaluation should account for the extent of calcific atherosclerosis and potential vascular access limitations—issues that are especially pronounced in CKD patients. Furthermore, contrast-induced nephropathy (CIN) is a concern in patients with advanced CKD. Nonetheless, minimizing contrast volume, ensuring adequate hydration, and using non-contrast imaging protocols when feasible are essential strategies to mitigate renal injury.

### 6.2 Outcomes and Prognosis

#### 6.2.1 Mortality

Across multiple large registry and meta-analysis studies, CKD and ESRD were consistently associated with higher short- and long-term mortality following TAVR. Gupta *et al*. [71] demonstrated a stepwise increase in in-hospital mortality, with rates of 3.8% in patients without CKD, 4.5% in CKD, and 8.3% in ESRD, corresponding to adjusted odds ratios (aOR) of 1.39 for CKD and 2.58 for ESRD. Similarly, Mohananey *et al*. [72] confirmed higher in-hospital mortality in CKD/ESRD cohorts compared with those without renal dysfunction. Meta-analyses have also corroborated these findings. Makki and Lilly [73] reported that advanced CKD significantly increased short-term (HR 1.51) and 1-year mortality (HR 1.56) in high-surgical-risk patients, whereas the association was not significant in low- to intermediate-risk groups. Wang *et al*. [74] pooled over 133,000 patients, demonstrating increased all-cause mortality at 30 days (RR 1.39), 1 year (RR 1.36), and 2 years (RR 1.20) among CKD patients. Patients on dialysis were at exceptionally high risk: Szerlip *et al*. [75] reported 1-year mortality of 36.8% in dialysis patients vs. 18.7% in non-dialysis patients, while Ogami *et al*. [76] observed 1-year mortality of 43.7% and 5-year mortality approaching 89%. Table 4 (Ref. [71, 72, 73, 74, 75, 76, 77, 78, 79, 80]) summarizes studies evaluating TAVR outcomes in patients with CKD/ ESRD.

**Table 4.** **Summary of studies evaluating outcomes of patients with CKD undergoing TAVR**.

Study (authors, year)	Design & population	Kidney disease definition	Outcomes assessed	Mortality	Other outcomes	Key findings
Gupta *et al*. (2017) [71]	NIS 2012–2014;	No CKD vs. CKD vs. ESRD	In-hospital mortality, MACCE/NACE	In-hospital mortality:	Major/Life threatening bleeding: CKD aOR ≈ 1.20; ESRD aOR ≈ 1.35	CKD/ESRD independently ↑ in-hospital death and complications after TAVR.
	Total 41,025			CKD aOR 1.39 (95% CI 1.24–1.55), ESRD aOR 2.58 (2.09–3.13); Mortality %: 3.8% (no CKD) vs. 4.5% (CKD) vs. 8.3% (ESRD).		
	No CKD 25,585		PPM, AKI		Vascular complications: aOR 1.15 (CKD), 1.25 (ESRD).	
	CKD 13,750					
	ESRD 1690				New PPM: aOR ≈ 1.08 (CKD), 1.12 (ESRD).	
					AKI ↑ in CKD; new dialysis rare but ↑ | Effect size: aOR ≈ 2.0 for AKI (CKD vs. no CKD).	
					LOS: Longer with CKD/ESRD.	
					Readmission rate: ↑ with CKD/ESRD.	
Mohananey *et al*. (2017) [72]	NIS 2011–2014;	No CKD/ESRD vs. CKD vs. ESRD	In-hospital mortality, LOS	Higher in-hospital mortality and adverse events in CKD/ESRD vs. no CKD.	Major/Life-threatening bleeding: ↑ with CKD/ESRD. Vascular complications: ↑ with CKD/ESRD. LOS: ↑ LOS with CKD/ESRD. AKI or new dialysis: ↑ LOS with CKD/ESRD. New permanent pacemaker (PPM): Similar to mildly ↑.	Renal dysfunction associated with worse in-hospital outcomes and longer LOS.
	Total 42,189;					
	No CKD 26,229;		PPM			
	CKD 14,252;					
	ESRD 1708					
Makki and Lilly (2018) [73]	Meta-analysis of observational studies reporting on advanced CKD and TAVR outcomes.	Advanced kidney disease	- Short-term mortality (in-hospital or 30-day)	Advanced CKD vs. No Advanced CKD	- Major bleeding: Increased risk in high-risk CKD patients (statistically significant); no significant association in low/intermediate-risk group.	Advanced CKD is significantly associated with higher short- and long-term mortality *and* increased bleeding and vascular complication risk—but only in high-surgical-risk TAVR patients. In low- to intermediate-risk patients, advanced CKD was *not* associated with significantly worse mortality or safety outcomes.
			- Long-term mortality (1-year).	High-risk patients:		
				Short-term mortality: HR = 1.51 (95% CI: 1.22–1.88), *p * < 0.01		
	11 studies included 10,709 patients.		Secondary: life-threatening bleeding, major vascular complications		- Major vascular complications: HR = 1.17 (95% CI: 1.01–1.35, *p * < 0.05) in high-risk CKD; not significant in low/intermediate group (HR = 1.11, 95% CI: 0.95–1.30, *p* = 0.07).	
				Long-term (1-year) mortality: HR = 1.56 (95% CI: 1.38–1.77), *p * < 0.01		
	Stratified by surgical risk: high-surgical-risk vs. low- to intermediate-risk					
				Low- to intermediate-risk patients:		
				- Short-term mortality: HR = 1.35 (95% CI: 0.98–1.84), *p* = 0.06 (not statistically significant)		
				- Long-term (1-year) mortality: HR = 1.08 (95% CI: 0.92–1.27), *p* = 0.34 (not significant)		
Lorente-Ros *et al*. (2024) [77]	NIS 2016–2020;	Normal renal function vs. CKD vs. ESRD	In-hospital mortality, cardiogenic shock/MCS, AMI, vascular, infection, respiratory, AKI, LOS	In-hospital mortality aOR:	Major/Life-threatening bleeding: CKD aOR ≈ 1.3; ESRD aOR ≈ 1.5.	Graded ↑ in death, bleeding; AKI in patients with CKD, pacemaker similar.
	Total 279,195;			CKD 1.4 (1.2–1.7)		
	Normal 187,325;			ESRD 2.4 (1.8–3.3)	Vascular Complications: CKD aOR ≈ 1.15; ESRD aOR ≈ 1.3.	
	CKD 81,640;			Mortality %: 1.1% (normal) vs. 1.6% (CKD) vs. 2.6% (ESRD).		
	ESRD 10,230				New PPM: Similar.	
					AKI or new ESRD: AKI aOR ≈ 5.0 (CKD vs normal);	
					Effect size: CKD aOR ≈ 5.0; LOS: ↑ with CKD/ESRD.	
Szerlip *et al*. (2019) [75]	STS/ACC TVT Registry 72,631 TAVR cases.	ESRD on dialysis vs. non-dialysis	In-hospital mortality; 1-year mortality; Bleeding and vascular complications	In-hospital mortality: 5.1% (ESRD) vs. 3.4% (non-dialysis).	Major/Life-threatening bleeding: ↑ ESRD (1.4% vs. 1.0%)	Dialysis status predicts markedly higher 1-yr mortality after TAVR.
	Total 72,631;			Major bleeding: 1.4% vs. 1.0%.	Vascular Complications: 1.2 × ESRD vs. non-dialysis	
	ESRD 3053 (4.2%)			1-year mortality: 36.8% (ESRD) vs. 18.7% (non-dialysis).		
					New PPM: Similar.	
					LOS: ↑ ESRD.	
Cubeddu *et al*. 2020 [79]	Retrospective sub analysis of PARTNER 1, PARTNER 2, and PARTNER 2 S3 randomized trials—	Baseline chronic kidney disease (CKD) stage ≥2 was present in 91% of participants	Change in CKD stage up to 7 days post-TAVR; post-TAVR eGFR; incidence of new dialysis; association between post-TAVR eGFR and intermediate-term survival	Overall, 30-day mortality after TAVR was 4.1%.	CKD Stage: Improved/Unchanged/Worsened	Among patients with severe aortic stenosis undergoing TAVR—even those with baseline impaired kidney function—CKD stage is more likely to remain stable or improve rather than worsen in the short term.
					- Stage 1: 77% improved or unchanged	
					- Stage 2: 90% improved or unchanged	
					- Stage 3A: 89% improved or unchanged	
	5190 patients				- Stage 3B: 94% improved or unchanged	
					- Stage 4: 99% improved or unchanged	
					Worsening to CKD Stage 5: 0.035% of patients (1 in 2892) progressed to Stage 5 within 7 days post-TAVR.	
					New Dialysis Requirement: 2.0% (70 of 3546 patients with available data) required temporary post-TAVR dialysis.	
Wang *et al*. (2022) [74]	Meta-analysis of 20 studies	Any CKD vs. no CKD; stage-stratified (3–5)	30 d/1 y/2 y all-cause mortality; 30 d AKI, Bleeding	30 d mortality RR 1.39 (1.31–1.47);	Major/Life-threatening bleeding: RR 1.33 at 30 d CKD vs. no CKD. Vascular Complications. AKI or new ESRD: RR 1.38 at 30 d (CKD vs no CKD) | Effect size: RR 1.38 (30 d; CKD vs. no CKD).	CKD increases early/mid-term mortality, AKI, bleeding. The higher the stage the higher the risk.
	133,624 TAVR patients			1 y RR 1.36 (1.24–1.49);		
				2 y RR 1.20 (1.05–1.38).		
				30 d AKI RR 1.38 (1.16–1.63);		
				30 d bleeding RR 1.33 (1.18–1.50).		
Kuno *et al*. (2020) [78]	Meta-analysis;	Dialysis vs. non-dialysis	Short-term and long-term mortality; Bleeding; PPM	Short-term mortality OR 2.18 (1.64–2.89);	Major/Life-threatening bleeding: OR 1.90 short-term (dialysis vs. non-dialysis) | Effect size: OR 1.90 (short-term). Vascular Complications: Similar to slightly ↑ | Effect size: Slight ↑ (NS pooled). New PPM: OR 1.33 ↑ | Effect size: OR 1.33.	Dialysis ≈ 2 × short-term mortality
	10 studies;					
	128,094 TAVR pts (5399 dialysis)			Long-term mortality OR 1.91 (1.46–2.50).		↑ bleeding/PPM.
Ogami *et al*. (2021) [76]	National cohort of ESRD patients undergoing TAVR (2011–2016).	ESRD on dialysis	30 d/1 y/5 y mortality	Mortality in ESRD: 5.8% at 30 d; 43.7% at 1 y; 88.8% at 5 y. 30 d mortality improved from 11.1% (2012) to 2.5% (2016).		Marked long-term mortality in ESRD despite improved peri-procedural metrics.
	n = 3883					
Allende *et al*. (2014) [80]	Multicenter TAVR cohort; advanced CKD (stages 4–5)	Advanced CKD; dialysis noted	Early/late mortality, bleeding; predictors	Dialysis HR 1.86 (95% CI 1.17–2.97) and AF HR 2.29 (1.47–3.58) predicted mortality; 1-year mortality as high as 71% when AF + dialysis coexisted.	Major/Life-threatening bleeding: ↑ with advanced CKD.	Advanced CKD = high-risk phenotype.
					AKI or new ESRD: High risk in stages 4–5; dialysis predicts death, LOS: ↑ with advanced CKD.	Longer hospital stay.

TAVR, transcatheter aortic valve replacement; CKD, chronic kidney disease; ESRD, end-stage renal disease; NIS, National Inpatient Sample; STS/ACC TVT, Society of Thoracic Surgeons/American College of Cardiology Transcatheter Valve Therapy; aOR, adjusted odds ratio; RR, relative risk; HR, hazard ratio; CI, confidence interval; LOS, length of stay; MACCE, major adverse cardiac and cerebrovascular events; NACE, net adverse clinical events; AKI, acute kidney injury; AF, atrial fibrillation. The upward arrow indicates an increase.

#### 6.2.2 Bleeding and Vascular Complications

Renal dysfunction was also associated with an increased risk of major bleeding and vascular complications. Gupta *et al*. [71] showed adjusted odds ratios of 1.20 and 1.35 for major bleeding in CKD and ESRD patients, respectively. Similarly, in a study by Lorente-Ros *et al*. [77], it was reported that there were graded increases, with aORs of 1.3 for CKD and 1.5 for ESRD. Data from meta-analyses also confirmed these trends: Wang *et al*. [74] found that CKD conferred a 33% increased risk of bleeding at 30 days (RR 1.33), while Kuno *et al*. [78] demonstrated nearly doubled short-term bleeding risk in dialysis patients (OR 1.90).

#### 6.2.3 Acute Kidney Injury and Dialysis Requirement

Acute kidney injury (AKI) was significantly more frequent among CKD patients. Gupta *et al*. [71] reported an approximate two-fold increase in AKI risk in CKD vs. no CKD, while Lorente-Ros *et al*. [77] found an adjusted odds ratio of ~5.0 for AKI in CKD compared with normal renal function. The requirement for new dialysis, although uncommon, occurred more frequently in CKD/ESRD cohorts. Cubeddu *et al*. [79] demonstrated that worsening renal function post-TAVR was rare, with 99% of stage 4 patients showing stable or improved kidney function and only 0.035% progressing to stage 5 within 7 days. However, 2% required temporary dialysis post-procedure [79].

#### 6.2.4 Readmission

Renal dysfunction is a strong predictor of early and late hospital readmissions following TAVR. In the Nationwide Readmissions Database, Gupta *et al*. [71] showed significantly higher 30-day readmission rates among patients with CKD and ESRD compared with those without CKD. An NRD analysis (2017–2018) demonstrated that ESRD patients had markedly higher 90-day all-cause readmissions (34.4% vs. 19.2%), with nearly two-fold increased hazard (HR 1.96, 95% CI 1.68–2.30), and increased cardiovascular readmissions (13.2% vs. 7.7%; HR 1.85, 95% CI 1.44–2.38) compared with non-ESRD patients [81].

#### 6.2.5 Other Clinical Outcomes

Permanent pacemaker (PPM) implantation rates were generally similar between CKD/ESRD and non-CKD groups, with only modest and inconsistently significant increases reported across studies [72, 75, 77, 78]. Hospital length of stay (LOS) was consistently longer among CKD and ESRD patients [72, 75, 77].

#### 6.2.6 Refinements in TAVR to Reduce Risk and Improve Outcomes in CKD/ESRD 

Newer generation valves, improvements in valve platforms, and refined procedural techniques have expanded the applicability and improved patient outcomes in high-risk populations like CKD patients [82]. Hydration strategies to prevent AKI in TAVR remain instrumental. Across randomized and observational studies, periprocedural hydration is consistently linked to lower rates of contrast-associated AKI after TAVR. In the single-center PROTECT-TAVI randomized trial, diuresis-matched hydration using the RenalGuard system reduced Valve Academic Research Consortium (VARC)-defined AKI from 25.0% with standard saline to 5.4% (*p* = 0.014), without any in-hospital dialysis and with no differences in 30-day mortality, cerebrovascular events, bleeding, or heart-failure hospitalizations [83]. By contrast, a more recent single-center randomized study in CKD patients (n = 100) reported no significant difference in post-TAVR AKI with RenalGuard versus standard pre/post-procedure hydration (21.3% vs. 15.7%; *p* = 0.651), and no differences in 30-day or 12-month mortality or complications, highlighting potential heterogeneity by setting, protocol, and patient selection [84]. Non-randomized pilot work in CKD cohorts suggested the benefit of RenalGuard for AKI prevention during TAVR, but these findings require confirmation in larger, contemporary randomized studies [85]. Outside of TAVR, high-quality randomized data support tailored hydration approaches that can inform TAVR practice. LVEDP-guided fluid administration (POSEIDON) lowered CI-AKI compared with a fixed-rate protocol after coronary catheterization (6.7% vs. 16.3%; RR 0.41; *p* = 0.005), with similar rates of dyspnea-related treatment discontinuation, indicating safety in volume-sensitive patients [86]. CVP-guided hydration in CKD/heart-failure patients undergoing coronary procedures likewise halved CI-AKI without increasing acute heart failure (15.9% vs. 29.5%; *p* = 0.006) [87].

#### 6.2.7 Zero-/ultra-low-contrast TAVR Workflows 

Case series and early feasibility reports show that TAVR can be planned and executed with little to no iodinated contrast by combining: non-contrast cardiac CT for calcium mapping, cardiac magnetic resonance (CMR) for annular sizing/coronary heights, duplex ultrasound for iliofemoral assessment, CO₂ angiography for access, and intraprocedural fluoroscopy/transesophageal echocardiogram (TEE) guidance (e.g., three-pigtail “cusp” technique) to establish the working view and deploy the valve. These protocols have been successfully used in severe CKD and prior CIN, with short hospital stays and no paravalvular leak reported in exemplar cases. While evidence is still limited to observational reports, the approach is feasible and specifically kidney-sparing [88, 89].

#### 6.2.8 Contrast Volume Limits Tailored to Kidney Function 

When contrast is used, several TAVR cohorts suggest indexing the dose to renal function. A contrast volume-to-estimated glomerular filtration rate (eGFR) ratio (CV/eGFR) ≥3.6 independently predicted both periprocedural AKI and 30-day mortality [90]; optimal cut-points for AKI prediction clustered around ~3.3–3.9 across studies. Earlier work proposed renal-function-based dosing to reduce AKI after TAVR, echoing PCI heuristics (e.g., “maximum contrast ≈ 3 × eGFR”). In practice, pre-specifying a patient-level ceiling (e.g., eGFR = 25 → ≤75 mL total) and tracking cumulative volume during the case are pragmatic safeguards [91].

#### 6.2.9 Prophylactic Dialysis/Hemofiltration

Outside of patients already on maintenance dialysis, routine “prophylactic” intermittent hemodialysis/hemofiltration (IHD)/heart failure (HF) to clear contrast is not recommended. Kidney Disease Improving Global Outcomes (KDIGO) advises against it, and randomized studies show no benefit—and potential harm—in patients with renal insufficiency [92, 93, 94].

## 7. Conclusion

TAVR presents a valuable option for the management of severe aortic stenosis in special patient populations that were historically underrepresented in pivotal clinical trials. These groups, including patients with active malignancy, psychiatric or neuropsychiatric disorders, and advanced organ dysfunction, face unique challenges. Yet, accumulating evidence suggests that TAVR can be performed safely with acceptable outcomes when appropriate patient selection and perioperative optimization strategies are implemented. Comprehensive multidisciplinary evaluation, careful risk stratification, and individualized decision-making that considers not only technical feasibility but also expected survival benefit and quality of life improvements are essential. Although short-term procedural outcomes are generally comparable to the broader TAVR population, long-term mortality may be higher in certain groups, primarily driven by non-cardiovascular causes related to underlying comorbidities. Further studies are warranted to refine protocols and improve safety and efficacy across these unique cohorts.
